# 4-E analysis and multiple objective optimizations of a novel solar-powered cogeneration energy system for the simultaneous production of electrical power and heating

**DOI:** 10.1038/s41598-023-49344-2

**Published:** 2023-12-14

**Authors:** Taufique Ahamad, Mohd Parvez, Shiv Lal, Osama Khan, Mohammad Javed Idrisi

**Affiliations:** 1 Department of Mechanical Engineering, Al-Falah University, Faridabad, Haryana 121004 India; 2 Department of Mechanical Engineering, Rajasthan Technical University, Kota, India; 3 Department of Mechanical Engineering, Jamia Millia Islamia, New Delhi, 110025 India; 4 Department of Mathematics, College of Natural and Computational Science, Mizan-Tepi University, Tepi, Ethiopia

**Keywords:** Environmental sciences, Environmental impact

## Abstract

Owing to its natural and rich advantages, exploration of solar energy technology has become increasingly popular in recent years to counter the growing crude oil prices. However, its universal adoption is still limited, not only due to environmental restrictions but also due to lower overall efficiency. Rankine cycle is optimised to conduct 4-E (Exergy, Energy, Economic and Ecological) analysis. Furthermore, three sets (R-113, R-11, and R-1233zd) of refrigerants are prioritised and ranked on the basis of 4-E analysis as outcomes. The contemporary study addressed all critical factors and explains the impact of solar irradiance, mass flow rate of molten salt and steam, turbine inlet pressure, and turbine inlet temperature which are eventually weighed and prioritised using combined multi-criteria decision making (MCDM) techniques. The energy efficiency, exergetic efficiency, power/ cost of electricity, and ecological emissions are taken as the indicators of the combined cycle, respectively. The energy efficiency of the hybrid system is improved to 75.07% after including cogeneration cycle, with an increment of 54.58%. In comparison to conventional thermal powerplant setups, the power/cost of electricity and ecological efficiency have been reduced by 68% and upgraded by 16%, correspondingly. Direct normal radiation is the most critical factor followed by turbine inlet temperature. Further, the result indicates that maximum exergy destruction that occurs in the central receiver declines to 39.92%, followed by heliostat and steam turbine which was 27% and 9.32% respectively. In conclusion, the hybrid cycle can furnish cheaper electricity, with lower carbon imprint in sustainable manner with better efficiency.

## Introduction

Recent years have witnessed an abrupt surge in population growth, thereby exerting excessive load on conventional sources of energy for power generation in powerplants^1^. This eventually has led to higher consumption of crude oil related sources, to an extent where researchers have prognosticated the exhaustion of current reserves by the year 2070^2^.

### Environmental concerns and health implications

These fossil run powerplants consumes substantial amount of different forms of power, where major part of the input supplied power is vanished into the atmosphere in the arrangements working on small-average temperatures surplus heat energy^3^. Such types of energy have extremely high potential and are potent contenders of effective power production. on the other hand, present coal-fired systems emit toxic and dangerous emissions which has been detrimental to the society^4^. Moreover, the generated effluents have the substantial potential to bind with host infection-based microbes such as a virus. The basic types of viruses developed in recent years which devasted the whole world were MERS, SARS, and COVID-19^5^. These viruses have strong affinity to bond with exhaust pollutants thereby resulting in being air-borne and increasing the chances of community spread^6^. To validate the above statement, a recent study probed and interlinked the daily deaths with growing pollution levels and daily deaths^7^.

### Solar energy as a sustainable solution

The growing pollution was a direct repercussion of the imposed restrictions being lifted which in evidently led to more lockdowns. Evolution of sustainable hybrid powerplants have motivated researchers to investigate and probe cleaner forms of energy available at different locations across the globe^8–11^. Due to the recent progress in the field of solar energy, researchers are extremely motivated to investigate the possible prospects of combined solar-thermal powerplants^12^. Location of India on world map provides a certain geographical advantage to the tropical country with substantial solar potential due to superior radiation intensity throughout the year. Subsequently, the mean solar radiation imparted is approximately close to 4.5–7.5 kWh/m^2^ with 310 sunlight potent days^13^. Around the globe, till date the current capacity of solar thermal powerplants ranges between 9277 and 10,020 Mwe^13^. The primary operation of such systems is to absorb any inherent heat into the working fluid from easily available turbine outlet interconnections. Such combined apparatus makes use of the waste heat issued out of the powerplant, in combination with solar energy to produce feasible energy^14^. Henceforth, such systems convert low-grade thermal energy from powerplants (heat energy) to produce high grade energy (mechanical work), rather than dumping high heat exhaust gases into atmosphere. Therefore, this practice safeguards the environment from unwanted heat energy^15^. Solar thermal powerplants (STP) seems to be clear winner since it facilitates superior power generation with lower running costs in a sustainable and cleaner manner. Henceforth identifying and designing the optimum input conditions for such cycles is of prime importance.

### Integration of solar energy and thermodynamic cycles

Organic Rankine cycle, Kalina cycle, Brayton cycle, Supercritical-CO_2_ hybrid cycle and cogeneration cycle have become the principal mode to deal with waste heat issued from coal-fired plants. However, the energy efficiency of such systems is limited^16–19^, which is primarily attributed to coal -fired powerplants input fuel cost in such cycles which is extremely high. In addition, individual efficiency of the system is lower. Integrating solar energy equipment’s with current set of thermodynamic cycles can improve the combined efficiency of the system and concurrently supress the exhaust pollutants issued from the plant. In the past decades, a large number of studies have been devoted to the development of heliostat field solar collectors integrated with solar receivers and placed on the solar tower^20–22^. This integrated system employs a working fluid primarily in molten salt. Furthermore, to counter the lower efficiency of the system a cogeneration cycle might be added to existing system for the simultaneous production of combined heating and electric power (CHP), thereby capable of increasing the overall efficiency of the system. From previous studies, it is also reported that a significant amount of heat energy in solar-powered plants is rejected through the steam turbine as a flue gas having a potent temperature of about 130℃. In past multiple scholars have investigated the outcomes of thermal powerplants. But potential of solar energy in such cycles needs to be probed further, so as to determine the best working cycle among others. For different sets of working fluids, the organic Rankine cycle (ORC) is primarily incorporated in systems to reintroduce small-average based temperature unused heat, while superheated carbon dioxide cycle is implemented to recover high-temperature waste heat^23^. Owing to its easy working, simple arrangement and enhanced consistency, ORC is extensively combined with solar equipment’s since low temperatures are produced by such integrated systems. multiple studies in past have considered customers demand over systems demand as paramount^24,25^.

**Figure 1:**
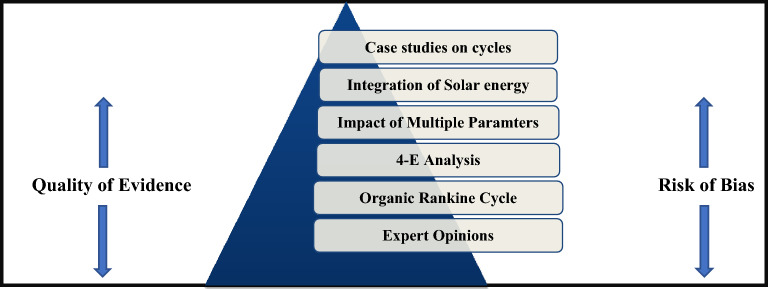
Hierarchy of Literature selection and operation parameters selection for the study.

The hybrid power block arrangements are examined in the past by incorporating supercritical CO_2_ Brayton cycles, advanced organic Rankine cycles, and other combined solar cycles^26,27^. Each of these arrangements display multiple benefits over orthodox designs on the basis of power, per unit electricity cost, exergy & energy efficiency and ecological efficiency. For the past few years researchers are heavily invested in probing multiple cycles capable of satisfying the above specified conflicting criterions. Identification of research gap is provideed by performing extensive literature survey. This procedure explores the present study bearing in mind several previous research publications, case studies, professional viewpoint etc. as displayed in the hierarchical Fig. 1.

Several researchers have found a few parameters which if regulated in a proper zone might create a rightful balance between reduction in energy consumption without hampering the original design^24–26^. Li et al. (2023)^33^ introduced an innovative organic-steam Rankine cycle, known as the partial cascade cycle, with the aim of enhancing both fluid evaporation temperature and thermal efficiency. According to their findings, this newly proposed system effectively raises the average temperature of the power fluid during the heating process, resulting in a remarkable maximum cycle efficiency of 45.3%. Gogoi et al. (2023)^34^ investigated a combined cycle power plant (CCPP) that integrates a gas turbine (GT) cycle, a regenerative steam turbine (ST) cycle, and a recuperative regenerative organic Rankine cycle (RR-ORC). The 3E analysis revealed a net power output of 54.22 MW, accompanied by energy and exergy efficiencies of 44.79% and 40.89%, respectively. The CCPP exhibited an overall system cost rate of 1965.43 $/h, with an exergoeconomic factor of 25.51%. Mahmoud et al. (2023)^35^ enhanced the performance parameters of a ground cooled ORC system. The capital cost and payback period ranges for the regenerative ORC were·17,062–25,592 and 3.7–5.5 years, respectively. Mirjavadi et al. (2022)^36^ presented a study to compare the two thermodynamic cascaded cycles using EES model, which consist of a steam cycle as a topping cycle and an organic Rankine cycle as a bottoming cycle. The obtained results showed that the Kalina cycle is superior one over the ORC cycle in a solar-driven steam Rankine cycle. In another study, Habibi et al. (2020)^37^ investigated a regenerative supercritical Brayton cycle using EES model, attached to an organic Rankine cycle at its bottom, and the cycle was driven by a solar-power tower applying molten salt as a working refrigerant. The result indicated that the maximum exergy efficiency was obtained at 21.23%, with net power output 177,321 of kW when helium is used as a refrigerant and the highest exergy destruction is to be obtained by 70,576 kW from oxygen.

### Research gap and objectives

**Figure 2:**
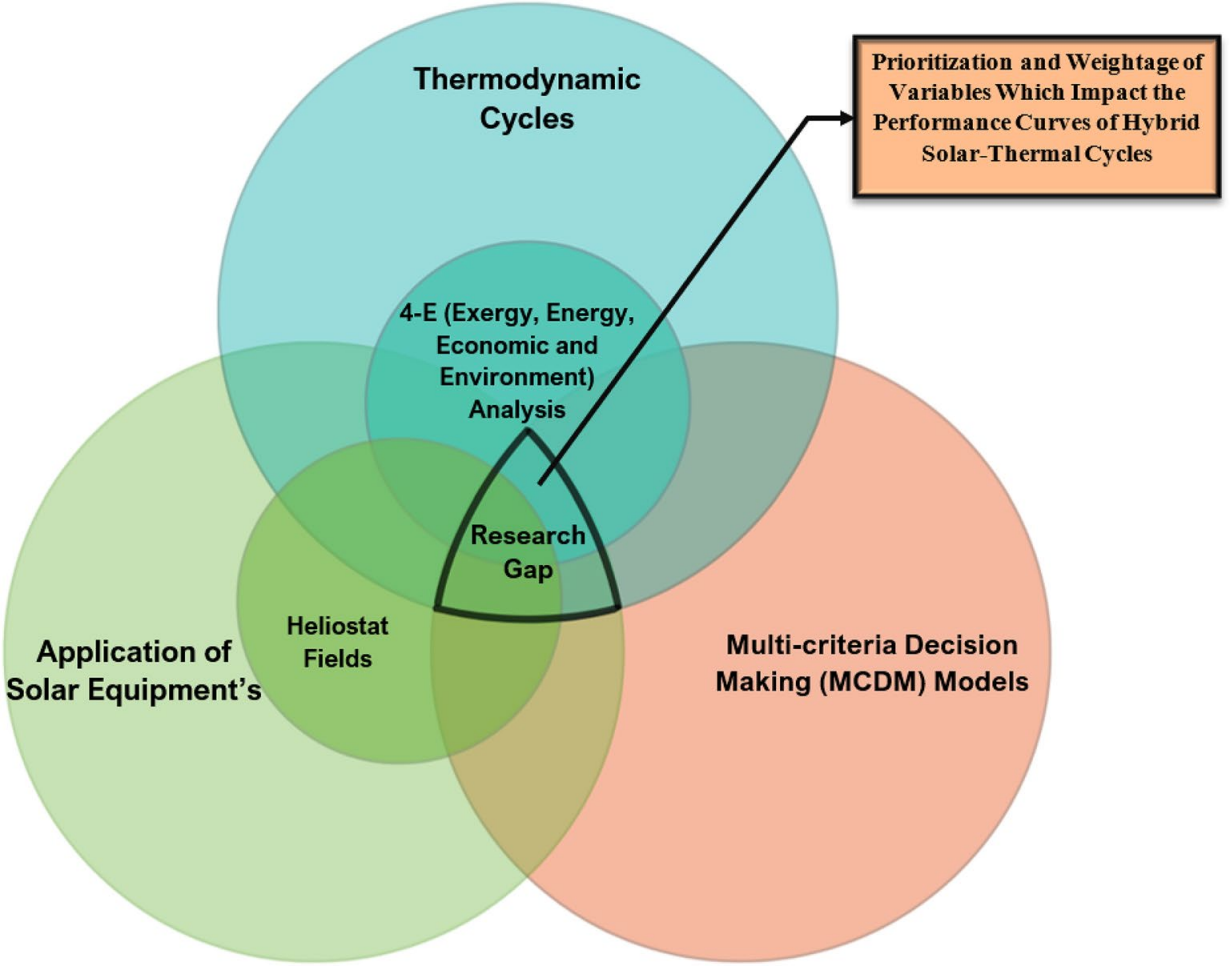
Research Gap addressed in this study.

The conclusions drawn from the literature shows that some essential aspects to solar thermal plants still remains unexplored as far as authors knowledge comprehends. Previous research in this field has predominantly focused on calculating exergy values for different equipment with varying input parameters. However, there has been limited attention given to prioritizing which specific parameter holds the greatest significance in influencing the overall outcomes of the system. The objective of this study is to address this gap by identifying and assessing the key parameters that have the most substantial impact on the collective performance of the system. By doing so, this research aims to provide valuable guidance to future power plant owners, enabling them to pinpoint and adjust specific parameters that will enhance the overall outcomes of their systems. The Fig. 2 shows the research gap of the study.

### Study methodology

The novelty of our study lies in the integration of the ORC cycle with the heliostat field's solar collector for combined heating and power generation in a solar cogeneration system, addressing research gaps in the literature. We introduce an innovative cascade solar-thermal arrangement to efficiently recover waste heat from the steam turbine, enhancing system performance. Additionally, our comprehensive analysis includes an in-depth thermodynamic investigation, economic analysis based on net electricity per unit, and assessment of the system's environmental impact. This holistic approach contributes to the knowledge base by providing new insights into the integration, performance optimization, economic feasibility, and environmental sustainability of solar cogeneration systems, thereby advancing the field of renewable energy and sustainable development.

It can be seen from the past available literature, that seldom studies have been reported on 4-E analyses by integrating the ORC cycle, heliostat field’s solar collector for combined heating and power generation. In order to recover the waste heat of steam turbine in a superior form, an innovative cascade solar-thermal arrangement is examined. In addition, the contemporary apparatus comprises of a heliostat field, organic Rankine cycle and cogeneration to produce electricity and thermal energy. ORC recuperates the surplus heat energy of steam turbine to produce more useful electrical energy. An enhanced thermodynamic investigation is executed in the contemporary research, while economic analysis is supplied with the aid of net electricity per unit. Effluents of the novel system on the atmosphere is also explored with ecological effectiveness feature as an indicator. A comprehensive parametric examination is executed, while prescribing weightages to each input parameter highlighting its significance in the Energy, exergy, economic, and environmental (4E) analysis. Multiple criteria decision making (MCDM) is used to rank the three working fluids i.e. R-113, R-11, and R-1233zd explored in the system based on best outputs of the system. Validation of the proposed setup is also provided so as to eliminate any adherent errors to enhance the accuracy of this analysis.

## System description and assumptions

The energy efficiency of the conventional integrated thermal powerplants is restricted evidently owing to the higher energy losses in the heliostats, collectors, boilers, elevated temperature outlet gas, and heat exchangers. Therefore, proper investigation of the contemporary system is performed by 4-E analysis of the proposed novel setup which might eventually effectively intensify the performance of the system. Current planned solar operated cogeneration energy system comprises of a steam Rankine cycle (RC), user heat, and organic cycle Rankine (ORC) with the aid of solar energy to generate combined heating and power.

**Figure 3:**
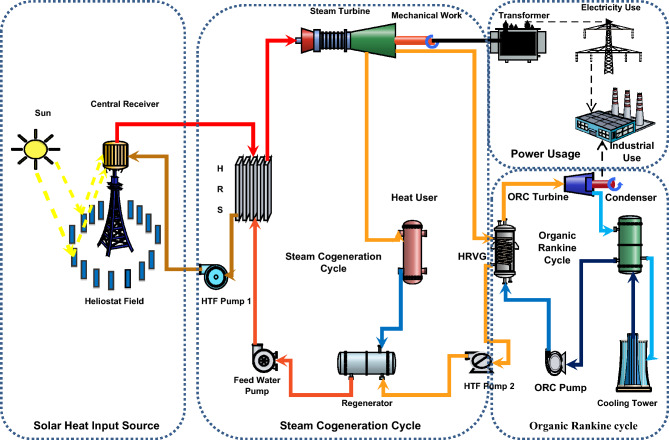
Schematic layout of the solar-ORC power plant.

Figure 3 offers a general outline of the entire integrated setup and the properties of a solar-operated cogeneration energy system are shown in Table 1. As far as the solar components are concerned it comprises of a set of heliostats that collects incoming radiations and further concentrates sunlight onto the receiver, which captures the focused sunlight and transfers the thermal energy of the radiations to a working fluid (molten salt). This heat transportation system, comprising of pipes, pumps, and valves, which facilitates heat transfer to power conversion systems. For effective combined solar-thermal systems the collection efficiency of the solar equipment is capped at 75%. The power transformation system comprises of a steam generator (HRSG), steam turbine, heat user, organic Rankine system, and supplies different types of apparatus which convert the thermal energy into electric power and produce the process heat in the heater.

**Table 1:** Properties of solar-operated cogeneration energy system.

System properties values		
Heliostat Field	Beam radiation (DNI) (mid value)	800 W/m^2^
	Overall field efficiency	75%
	Total heliostat aperture area	10,000 m^2^
Central Receiver	Aperture area	12.5 m^2^
	Inlet temperature of molten salt	290 °C
	Outlet temperature of molten salt	565 °C
	View factor	0.8
	Tube diameter	0.019 m
	Tube thickness	0.00165 m
	Emissivity	0.8
	Reflectivity	0.04
	Wind velocity	5.0 ms^−1^
	Passes	20
Steam generator	The internal temperature of water	240 °C
	Outlet temperature of steam	550 °C
	Ambient temperature	20 °C
Organic rankine cycle	Working fluid used in ORC	R-113, R-11, R-1233zd
	Mass flow rate of waste heat source	1 kg/s
	ORC turbine inlet pressure (bar)	15–35 (range)
	ORC turbine outlet pressure (bar)	0.5
	ORC turbine isentropic efficiency	80%
	ORC pump isentropic efficiency	75%
Thermo—Physical Properties of Molten Salt	Temperature range of molten salt	220–574 °C
	Viscosity of molten salt	25 (MPa-s)
	Density of molten salt	863(Kg/m^3^)
	Thermal conductivity of molten salt	0.134(W. K^−1^.m^−1^)
	Specific heat of molten salt	10(kJ. K^−1^.kg^−1^)
	Freezing temperature of molten salt	238 °C

In the cogeneration energy system, the waste heat of steam obtained at the exit of the steam turbine moves into the heat exchanger which is called heat recovery vapour generation (HRVG) where it transfers its remaining heat to recover the energy and runs the ORC system^37^. Further, the refrigerant transpires from the HRVG, where the heated organic vapour enters the refrigerant turbine and produces the power on its expansion which might be transferred to a nearby housing community^38^. Furthermore, this thermal energy is transferred into the atmosphere within the ORC condenser, enabling lowered temperature refrigerant into the heat exchanger to complete the cycle^39^. Stream composition data of input and outputs of different components for cogeneration energy system shown in Table 2. In the combined solar-thermal plant, heliostat field and solar receiver constitute roughly half of the installation price of the system for operations producing medium–high power^25^. Therefore, the thermal power block (ORC and cogeneration system) parameters need to be carefully designed so as to achieve zenith efficiency at lower initial and operating cost. Also, direct normal irradiation estimation and collection is an essential parameter which needs to be considered in the analysis, since it is the prime source of energy generation. In prior literature, the thermal-economic analysis established that ORC systems are capable of working effectively with appreciable performance for a substandard thermal equipment set also. Moreover, opting for suitable working fluid for the ORC system also impacts the power generation process. Henceforth it is essential to investigate the operating conditions of hybrid plants by undertaking different working fluids and achieving a non-conflicting thermo-economic-environment solution. The choice of working fluid in ORC system is based on the results previous study^41^. The study states that solar thermal heat available at 250 °C, R-11 is the one of the most reasonable options for ORC as refrigerant at the specified power capacity. The major disadvantage with solar setups is the inconsistency in the power generation process due to unpredictable weather conditions. Due to this the efficiency of the combined system decreases drastically at part loads. In order to keep the plant running at optimum conditions, the plant would shut itself down, when energy input from the sun is less than 60% of the thermal load. Therefore, it is necessary to pre-analyse the variables which impact the performance of the plant so as to keep the plant in running condition as much as possible during the daytime^56^. Depending on the quantity of thermal energy produced by the solar field, the transformed mechanical energy is stored in the form of electrical energy in the transformer which later distributes the power to a nearby residential community^57^.

**Table 2:** Stream composition data of input and outputs of different components for cogeneration energy system.

State point	Pressure (bar)	Temperature (°C)	Flow rate (kg/sec)	Specific enthalpy (kJ/kg)	Specific entropy (kJ/kg K)
1	225	565	12.32	1580.42	7.80
2	178	290	12.32	627.80	6.98
3	178	290	12.32	627.80	9.98
4	160	500	1.794	3297.10	6.305
5	32	255	1.794	2876.56	6.380
6	32	500	1.794	3454.00	7.203
7	16	421.46	1.794	3303.22	7.335
8	16	188.20	1.794	2363.16	5.539
9	16	102.60	1.794	858.60	2.342
10	16	103.90	1.794	873.62	3.747
11	0.10	100	1.794	2324.40	7.335
12	0.10	100	1.794	193.29	0.649
13	16	102.50	1.794	193.78	0.649
14	25	77.52	0.1547	429.32	1.6895
15	0.5	52.12	0.1547	373.06	1.6895
16	0.5	42.53	0.2583	148.57	0.64
17	25	47.19	0.1035	100	0.64

## Methodology

### Model validation

Since, there is no such thing as a perfect cycle. All hybrid cycles have errors and uncertainties, no matter how hard we might try to minimize them. The primary reasons for taking out the uncertainties in the proposed model is to validate the hybrid combination of cycles provided in the research by comparing it with previous cycle results. The combined output of the system may vary significantly than individual cycles. Henceforth, the current section explores exergy losses in the system and has taken it into consideration. Errors and uncertainties may occur during the measurement of climatic conditions, calibration methods, method of observation, and testing in the solar power cogeneration system integrated with heliostat field system, steam turbine cycle, heat user, and organic Rankine cycle system, and global solar radiation at the regular interval of time. The cogeneration setup considered in the system have been justified in one of the prior studies^33^. Specific outcomes evaluated developed a difference for energy efficiency of the cogeneration model as 1.15% in comparison to previous model^41^. While considering the Rankine system, the outcome thermal efficiency is estimated at an average difference of 2.19% in reference to previous cycle^41^. Consequently, the only validation required, is in solar energy equipment arrangement in the analysis. Comparative outcomes attained for solar based apparatus with Reference^39,42^ are displayed in Table 3. The zenith difference transpires in the heliostat field, with a mean difference of 1.05%. The outcomes illustrated that the combined solar arrangement has decent and achievable agreement with prior models.

**Table 3:** Measurement accuracies and experimental uncertainties associated with sensors and parameters.

S. No	Sensors and parameters	Accuracies and uncertainties measurement
1	T-type thermocouples	± 0.3 C
2	Flow meter	± 0.04 ml
3	Pressure transducer	± 0.2 mbar
4	Voltage measurement	± 0.1 V
5	Current measurement	± 0.1 A
6	Power temperature coefficient	± 0.3%/C

The total uncertainties evaluating equations in the generalized form have been taken into account in the calculation for individual variables^38,40^ given below:

Consider a general case in which an experimental result r, is a function of j measured variables X_i_; than

1 $$ r = r\left( {X_{1} ,~X_{2}  \ldots  \ldots  \ldots  \ldots .,X_{j} } \right) $$

The above equation is the data reduction equation used to determine the measured values of the variables X_i_. Then the uncertainty in the result is given by Holman as;

2 $$ U_{r}  = \left[ {\left( {\frac{{\partial r}}{{\partial X_{1} }}} \right)^{2} .~U_{{x1}}^{2}  + \left( {\frac{{\partial r}}{{\partial X_{2} }}} \right)^{2} .~U_{{x2}}^{2}  +  \ldots  \ldots  \ldots  \ldots  \ldots  \ldots \left( {\frac{{\partial r}}{{\partial X_{j} }}} \right)^{2} .~U_{{xj}}^{2} } \right]^{{1/2}}  $$

where ${U}_{xj}$ are the uncertainties in the measured variable X_i_. The calculation of experimental uncertainties carried out which are shown in Table 3 as follows:

The overall percentage of error was estimated during the experimentation using Eq. (3).

3 $$ U = ~\left( {\left[ {\frac{{\partial R}}{{\partial x_{1} }}W_{1} } \right]^{2}  + \left[ {\frac{{\partial R}}{{\partial x_{2} }}W_{2} } \right]^{2}  + ~ \ldots  \ldots .~ + ~\left[ {\frac{{\partial R}}{{\partial x_{n} }}W_{n} } \right]^{2} } \right)^{{{\raise0.7ex\hbox{$1$} \!\mathord{\left/ {\vphantom {1 2}}\right.\kern-\nulldelimiterspace} \!\lower0.7ex\hbox{$2$}}}}  $$

The overall percentage of uncertainty (U) = square root of [(Error level in Heliostat field value)^2^ + (Error level in DNI collection value)^2^ + (Error level in DNI prediction value)^2^ + (Error level in solar receiver value)^2^]^1/2^.

The overall percentage uncertainty in solar equipment = Square root of [(1.57)^2^ + (0.52)^2^ + (0.81)^2^ + (1.2)^2^]^1/2^ = 2.19%

The overall percentage of uncertainty in the combined setup =  ± 2.19% + 1.15% + 1.05% = 4.39%

The total uncertainty level for the combined setup is estimated to be ± 4.39%, thereby lying-in acceptable range.

### Working fluid selection

Table 4 summarizes the comparative thermophysical properties of working fluids such as R11, R113, and R1233zd. The fluids considered have zero ozone depletion potential (ODP) and negligible global warming potential (GWP). The selection of these fluids is based on the operating properties such as enthalpy and temperature values of the hybrid system at various points, also validated by previous studies^41,42^. The selection of refrigerants, such as R-113, R-11, and R-1233zd, in the study of solar cogeneration within a Rankine cycle is significant for multiple reasons. Firstly, these refrigerants exhibit excellent thermal stability, enabling them to withstand the high temperatures and pressures encountered in the Rankine cycle. This characteristic ensures efficient and reliable operation of the system. Secondly, the environmental impact is a crucial consideration, and R-113, R-11, and R-1233zd have low ozone depletion potential (ODP) and negligible global warming potential (GWP). By using these refrigerants, the study aligns with the goal of reducing greenhouse gas emissions and preserving the ozone layer. Additionally, the thermodynamic properties of these refrigerants contribute to optimizing the system's overall efficiency. Their specific heat capacities, boiling points, and thermal conductivities play a vital role in achieving optimal energy conversion and heat transfer within the Rankine cycle. Lastly, the safety aspect is paramount, and R-113, R-11, and R-1233zd possess favorable safety characteristics such as low toxicity levels and non-flammability, ensuring the safety of both the system and its operators. Therefore, the choice of these refrigerants provides advantages in terms of thermal stability, environmental impact, efficiency optimization, and safety considerations, making them suitable for studying solar cogeneration in a Rankine cycle.

**Table 4:** Thermophysical properties of working fluids.

Fluids	Name	Critical temperature (^o^C)	Critical pressure (bar)	Melting point (^o^C)	Boiling point (^o^C)	Molecular weight (g/mol)	Global Warming Potential (CO2e)	Ozone Depletion Potential (ODP-Units)
R11	Trichloromonofluoromethane	198.11	43.94	−110.48	23.77	137.37	4500	0
R113	1,1,2-Trichloro-1,2,2- Trifluoroethane	214.21	33.92	−35	47.59	187.38	4750	1
R1233zd	Trans-1-Chloro-3,3,3-trifluoropropene	165.6	35.73	−78	18.31	130.50	< 1	0

### Mathematical modelling

To investigate the performance of the hybrid setup, a complete mathematical illustration is developed comprising of several equations which undertakes energy investigation, exergy investigation, environmental investigation, and economic investigation for the novel system. Some basic assumptions were formulated in the study and are explained below:

- The complete model is an integration of different components aligned together to work as a single model and is further presumed to work at the steady-state condition with constant solar insolation.
- During the entire analysis, the ambient conditions in which the model worked assumed a standard and constant value of temperature T_0_ and pressure p_0_ which are 20 °C and 1 bar.
- Subsequent pressure drops and thermal losses into the atmosphere for the majority of the apparatuses of the model are not taken into consideration.
- Sudden variation or alterations in the potential and kinetic energies are not taken into the formulations of this study.
- The chemical exergy associated with the materials is not taken into consideration.

#### Thermodynamics analysis

Analysis of energy and exergy analysis has been carried out for mass, energy, and exergy balances which enables in pinpointing the exact location within the model whose components have maximum thermodynamic inefficiencies.

- Energy efficiency of heliostat (H)

The heliostat field is an integration of several stacked-up heliostats representing and focusing the majority of the sun rays on the centralized dedicated receiver. Since the rate at which a major portion of solar thermal input is being received may be written as:

4 $${\dot{Q}}_{solar}={A}_{field}q$$

where q is the number of solar radiations received per unit area and (A_field_) is the countable area of the heliostat field this is linked to the field of opening (A_app_) in terms of concentration ratio written as:

5 $$C=\frac{{A}_{{\rm{field}}}}{{A}_{{\rm{app}}}}$$

The central receiver receives half of the thermal energy obtained from the heliostat, and the remainder is lost to the atmosphere and is written as:

6 $$\dot{Q}_{{{\rm{solar}}}} = \dot{Q}_{{{\rm{CR}}}} + \dot{Q}_{{\rm{lost, heliostat}}}$$

Heliostat's energy efficiency can be evaluated from the following equation:

7 $$\eta_{{\rm{energy, heliostat}}} = \frac{{\dot{Q}_{{{\rm{CR}}}} }}{{\dot{Q}_{{{\rm{solar}}}} }}$$

- Energy efficiency of central receiver (CR)

The thermal energy from the central recipient which is consequently drained into the atmosphere by molten salt and remainder is lost are evaluated by the following equations:

8 $$\dot{Q}_{{{\rm{CR}}}} = \dot{Q}_{{{\rm{moltensalt}}}} + \dot{Q}_{{\rm{lost, CR}}} = \dot{m}_{{{\rm{moltensalt}}}} \left( {h_{2} - h_{1} } \right) + \dot{Q}_{{\rm{lost, CR}}}$$

9 $$\eta_{{\rm{energy, CR}}} = \frac{{\dot{Q}_{{{\rm{moltensalt}}}} }}{{\dot{Q}_{{{\rm{CR}}}} }}$$

- Energy efficiency of entire system

It is the ratio of useful energy produced in the entire system to the input energy of the fuel supplied to the entire system, can be written as:

10 $$\eta_{{{\rm{energy}}}} { = }\frac{{\dot{W}_{ST} + \dot{W}_{RT} - \dot{W}_{HTF,P1} - \dot{W}_{HTF,P2} - \dot{W}_{FW,,P3} - \dot{W}_{ORC, P4} - \dot{W}_{c} , ORC}}{{\dot{Q}_{C.R} }}$$

#### Exergy efficiency of entire system

The product exergy output is known as the exergy input separated into the whole system and it can be evaluated by the following equation:

11 $$\eta_{{{\rm{exergy}}}} = \frac{{\dot{W}_{ST} + \dot{W}_{ORC,T} - \dot{W}_{HTF,P1} - \dot{W}_{HTF,P2} - \dot{W}_{FW,P3} - \dot{W}_{ORC, P4} - \dot{W}_{c} , ORC}}{{\dot{E}_{x,in} }}$$

Exergy signifies the zenith useful work achieved from the proposed cycle for multiple ranges of temperature and pressure for the circulating substance with reference to ambient atmospheric settings. In addition, such thermodynamic examinations are also capable of deriving the losses or destruction rate in each respective module of the apparatus. Any equipment undergoing substantial losses or higher exergy destruction is considerably deviating from ideal conditions requires surplus examination to optimize its input parameters. For this reason, kinetic and potential exergies might be ignored for the analysis. To further validate the process, comprehensive form of energy and exergy balance equations of each component for the solar-powered cogeneration energy system are briefed in Supplementary Section Tables A1 and A2.

#### Environmental analysis

For any analysis to be complete, it is essential to investigate, the overall environmental impact of the setup working under different climatic conditions. Although the contemporary plant uses only natural resources for energy generation, however there is still some exhaust effluents released into the environment which might be detrimental to the ecology of the planet. The current formula effectively exemplifies and identifies the link amongst power generation apparatus and environment, ecological efficiency (EE), has been explored in the current study by investigating the environmental performance of power setup ^41^.

12 $${\rm{EE}}={\left[0.204{\eta }_{e}\,{\rm{ln}}(135-{\rm{IP}})/({\eta }_{e}+{\rm{IP}})\right]}^{0.5}$$

13 $${f}_{{CO}_{2}e}={f}_{{CO}_{2}}+80{f}_{{SO}_{2}}+50{f}_{{NO}_{x}}+67{f}_{PM}$$

where, ${f}_{{CO}_{2}e}$ is the Carbon dioxide equivalent emission coefficient.

14 $${f}_{{CO}_{2}}^{CO}=985\times {10}^{-3}LHV/10466$$

15 $${f}_{PM}=\left({C}_{PM}\times {10}^{-6}\right){V}_{vg}/{m}_{steam}$$

16 $${f}_{{NO}_{x}}=\left({C}_{{NO}_{x}}\times {10}^{-6}\right){V}_{vg}/{m}_{steam}$$

17 $${f}_{{SO}_{2}}=\left({C}_{{SO}_{2}}\times {10}^{-6}\right){V}_{vg}/{m}_{steam}$$

where, $${C}_{PM}=1(mg/N{m}^{2})$$, $${C}_{{NO}_{x}}=148 (mg/N{m}^{2})$$, $${C}_{{SO}_{2}}=5.44 (mg/N{m}^{2})$$, $${{\rm{V}}}_{{\rm{vg}}}\text{ is volume flow rate of exhaust gas},\text{ in N}{{\rm{m}}}^{3}/{\rm{s}}$$

where η_e_ and IP are the energy efficiency of hybrid setup and pollution gauge. Furthermore, the EE factor ranges between 0 and 1. In addition, greater EE is, better as it depicts healthier and cleaner impact on the atmosphere of the system.

#### Economic analysis

To estimate the economic performance of any plant from lifetime viewpoint, levelized cost of energy (LCOE) is a capable tool applied to characterize the average cost of per electricity ^26^. Frequently, used unit for the above parameter is expressed in terms of $/kW. Recent studies have established that the LCOE of electricity, heating and cooling are alike. Generally, the universal common formula of LCOE is evaluated as,

18 $${\rm{LCOE }} = {\rm{ C}}_{{{\rm{total}}}} / \, \left\{ {{\rm{W}}_{{{\rm{net}}}} \times \, \tau } \right\}$$

where C_total_ is the total cost for the energy system and W_net_ is the annual total net power output, including electricity, heating and cooling. τ is the lifetime of the energy system.

### Operating parameters along with their ranges

The combined ORC-Cogeneration plant is powered by a low-grade natural heat source such as solar energy which has lower temperatures as compared to conventional steam powerplants. The turbine inlet pressure (TIP), turbine inlet temperature (TIT), turbine exit pressure (TEP), mass flow rate of molten salt (ṁ) and Direct normal irradiation (DNI) are selected as the input variables which needs to be varied to explore the impact on outcomes of the cycle. The parameters Turbine Inlet Pressure (TIP), Turbine Inlet Temperature (TIT), Turbine Exit Pressure (TEP), Mass Flow Rate of Molten Salt (ṁ), and Direct Normal Irradiation (DNI) play crucial roles in the study of solar cogeneration in a Rankine cycle. Following parameters are shown below:

- Turbine Inlet Pressure (TIP): TIP determines the pressure at which the working fluid enters the turbine. It affects the power output and efficiency of the turbine. Optimizing TIP helps achieve the desired power generation and maximize energy conversion in the Rankine cycle.
- Turbine Inlet Temperature (TIT): TIT signifies the temperature of the working fluid as it enters the turbine. Higher TIT results in higher energy extraction from the heat source and improves the overall efficiency of the cycle. Accurate control and understanding of TIT are essential for optimizing the performance of the system.
- Turbine Exit Pressure (TEP): TEP refers to the pressure at which the working fluid exits the turbine. It affects the back pressure on the turbine and influences the expansion process. Proper selection and control of TEP are necessary to balance turbine efficiency, power output, and overall system performance.
- Mass Flow Rate of Molten Salt (ṁ): The mass flow rate of molten salt represents the quantity of heat transfer fluid circulating in the solar receiver. It directly affects the amount of thermal energy absorbed from the solar source and subsequently impacts the power generation capacity of the Rankine cycle. Accurate estimation and control of ṁ are crucial for system performance and efficiency optimization.
- Direct Normal Irradiation (DNI): DNI refers to the solar radiation incident on a surface perpendicular to the sun's rays. It represents the availability of solar energy for the system. Understanding the DNI helps in sizing and designing solar collectors, predicting energy output, and assessing the feasibility of solar cogeneration projects.

Overall, these parameters significantly influence the performance, efficiency, and power output of the solar cogeneration system in a Rankine cycle. Proper control and optimization of TIP, TIT, TEP, ṁ, and DNI are essential for achieving maximum energy conversion, improving system efficiency, and ensuring the viability of solar cogeneration projects.

Molten salt is considered as the heat transfer fluid that absorbs and delivers the necessary heat from the solar receiver into the heat recovery system owing to its high thermal conductivity. Further, steam created is taken into the turbine from the recovery system and converted into mechanical work. The advantage of high specific heat results in the decreases of the thermal losses in form of exergy. The turbine inlet temperature is the temperature difference between the temperature at the entry of turbine in the form of steam and exit of the solar receiver in the form of molten salt the other fluid. The turbine inlet temperature is ranged between 400 and 600 ℃ based on previous successful experiments. The atmospheric conditions assumed in this study were 25℃ and 1 atm. To understand the impact of essential variables on the 4E outcomes, six variables were considered in the study. These variables are specially designed and chosen as they strongly influence the performance of the system^44,45^. The values for the above-mentioned operating variables are presented in Table 5. The hybrid apparatus has been designed for 4000 operational hours for continuous operation.

**Table 5:** Operating parameters along with their levels.

S.No	Operating parameter	Designation	L1	L2	L3	L4	L5
1	Turbine inlet pressure (TIP)	A	180	190	200	210	220
2	Turbine inlet temperature (TIT)	B	400	450	500	550	600
3	Turbine exit pressure (TEP)	C	5	10	15	20	25
4	Mass flow rate of molten salt (ṁ)	D	1.5	1.8	2.1	2.4	2.7
5	Direct normal irradiation (DNI)	E	600	700	800	900	1000
6	Refrigerant		R11		R113	R1233zd	

### Multiple criteria decision making (MCDM)

It is essential to understand and contemplate the impact of each variable on the system by analysing their importance since the effect or degree of each parameter might be diverse. On the basis of the analytical hierarchy process (AHP) prioritization outcomes, we categorize that selected set of specific input constraints hold higher impact on the system performance while the other variables variation might be insignificant to the system. The current study explores four objective functions energy, exergy, economic and ecological impact. Often with multi-criteria problems it is seen that if a single objective function attains ideal solution, generally another objective function might not reach to its potential, specifically in case of problems where pareto-ideal conditions of these 2 outcomes is inverse. Combined AHP-TOPSIS analysis delivers best possible outcomes for the applied functions by varying inputs.

#### Analytical hierarchy process (AHP) methodology

The present AHP technique was initially introduced by Saaty with the main goal of developing a novel process of analysing the weight of multiple criterions by a relative measurement method purely based on qualitative and rational ideologies. It is a famous tool earlier used in multiple researches for classifying multiple aspects based on this judgemental tool. In other words, it can also be seen as a conflict solving technique where optimisation of multiple outcomes might result in irregularity in the problem statement. Therefore, the particular technique permits the problem statement to signify multiple weights to different criterions, thereby maintaining the regularity and reliability of the problem statement.

The subsequent problem solved by AHP is explained below in multiple steps: -

*Step 1* In the primary stage, we need to recognize the objectives needed to achieve, eventually bringing the number of choices down to below required number.

Classifying constraints and judgement them built on a computable feature necessitates applied proficiency of an experienced skilled persons who can existing contemporary a appropriate clarification and arrangement of the accessible possibilities.

*Step 2* A Through valuations requirements to be accomplished, which off the record into two segments: (i) Amongst Characteristics of the problem (ii) Amongst numerous Substitute results that accomplish each requirement.

Specialists of the compulsory arena have established matrices which investigates characteristics on a prime scale extending between 1 and 5.

The amount of interconnected vectors is premeditated by means of the formulation (nxn), where n stipulates the amount of characteristics.

*Step 3* Let Xij specifies the ith factor's importance position in judgement to the jth factor. Afterwards that, Xji = 1/X_ij_.

*Step 4* Formulation of a consistent pair-wise medium which is formed for the functional arrangement in subsequent sub-steps: -

- Estimate and formulate the over-all sum of all columns.
- Remove the resultant columns from the sum to each element of the matrices.
- For receiving the comparative weights, estimate the amount of rows.

*Step 5* Application of Eq. (1), the Evaluation matrix I attained, peak Evaluation outcome, and Criterion index (CI)

19 $$CI=\frac{\lambda \mathit{max}-n}{n-1}$$

The Eigenvector of the conjoined analysis matrices is zenith, and the amount of parameters are n.

*Step 6* Reapplying Eq. (1), the consistency ratio (CR) is evaluated (2).

where RI means random index,

20 $$CR=\frac{CI}{RI}$$

#### TOPSIS assignment

The tables extracted for weight determination are further transferred to prioritization model such as TOPSIS. This technique further validates the energy based criterions so as to priority ranking for different set of problems. It includes of subsequent phases in developing feasible prioritization model:

*Step 1* Collect all possible information pertaining to doctors for ranking prioritization process concerning the vital assessments of the consequence answers in linguistic relationships such as extremely low, low, average, high, extremely high given by analyst. Clearly define the decision problem and the criteria that will be used to evaluate the alternatives.

*Step 2* Create a decision matrix where each row represents an alternative and each column represents a criterion. Assign numerical values to each alternative based on their performance on each criterion. Converting numerous linguistic principles into numbers.

$${\rm{X}}_{{{\rm{abN}}}} = \left( {{\rm{ l}}_{{{\rm{abN}}}} , \, , \, } \right){\rm{ Where}},{\rm{ a }} = { 1},{ 2},{ 3}.........{\rm{ m}};{\rm{ b }} = {1},{ 2},{ 3}......... {\rm{n}},$$

where,

21 $$a={\rm{min}}\left\{{l}_{abN}\right\}, b=\frac{1}{N}\sum_{N=1}^{N}{P}_{abN }, c={\rm{max}}({u}_{abN})$$

*Step 3* Assign weights to each criterion based on its relative importance. The weights should add up to 1. Multiply each element in the normalized decision matrix by its corresponding weight. Estimate the consequence answers for the collective weights.

22 $$B= {\left[{P}_{ij}\right]}_{mxn}$$

Here, i = 1, 2, 3, …., m; j = 1, 2, 3, …., n

23 $${P}_{ij}= \left(\frac{{a}_{ij}}{{c}_{j}^{*}}\right); {c}_{j}^{*}={\rm{max}}\,{c}_{ij}$$

24 $${P}_{ij}= \left(\frac{{a}_{j}^{-}}{{c}_{ij}} \right); {a}_{j}^{*}={\rm{min}}\,{a}_{ij}$$

*Step 4* Normalize the global output matrices.

25 $$V= {\left[{v}_{ij}\right]}_{mxn} where, i=1, 2, 3, \dots , m; j=1, 2, 3, \dots , n$$

26 $$Here, {v}_{ij}= {p}_{ij} \left(\times \right){w}_{j}$$

*Step 5* Figure out the homogenous weighted matrices.

27 $${A}^{+}= \left\{{v}_{1}^{+} ,\dots \dots .., {v}_{n}^{+} \right\} Where,$$

28 $${v}_{j}^{+}= \left\{{\rm{max}}\left({v}_{ij}\right)IFj \in J; {\rm{min}}\,{v}_{ij}\,IF\,j\in J^{\prime}\right\}, j=1, 2, 3, \dots , n$$

$${A}^{-}= \left\{{v}_{1}^{-} ,\dots \dots .., {v}_{n}^{-} \right\} Where,$$

29 $${v}_{j}^{-}= \left\{{\rm{max}}\left({v}_{ij}\right)IFj \in J; {\rm{min}}\,{v}_{ij}\,IF\,j\in J^{\prime}\right\}, j=1, 2, 3, \dots , n$$

*Step 6* For each criterion, determine the maximum and minimum values across all alternatives in the weighted normalized decision matrix. The vector of the maximum values, is the vector of the minimum values. These vectors represent the ideal solutions in terms of the criteria. Establish the optimal solutions that are both positive and negative.

30 $${d}_{i}^{+}= {\left\{\sum_{j=1}^{n}\left({v}_{ij}-{v}_{ij}^{+}\right)\right\}}^{1/2}; i=1, 2, \dots , m$$

31 $${d}_{i}^{-}= {\left\{\sum_{j=1}^{n}\left({v}_{ij}-{v}_{ij}^{-}\right)\right\}}^{1/2}; i=1, 2, \dots , m$$

*Step 7* Calculate the relative closeness of each alternative to the ideal solution by dividing the distance by the sum of the distance to the for each alternative. Calculate the variances amongst the definite data collected from optimum both positive and negative.

32 $$C{C}_{i}= \frac{{d}_{i}^{-}}{{{d}_{i}^{-}+d}_{i}^{+}};i=1, 2, \dots , n$$

*Step 8* Approximate the ultimate value of closeness coefficient (CC) and simultaneously approximate the prior investigational values based on them, preliminary with the study pertaining to the zenith CC value manifested with maximum rank. The rank reduces with decreasing CC value. Rank the alternatives based on their relative closeness values. The alternative with the highest relative closeness is considered the best choice.

Table 6 demonstrates the linguistic peers allocated to the output values.

**Table 6:** Quantified values assigned to the outcome reactions of the significance levels.

S.No	Outcomes	Turbine inlet pressure (TIP)	Turbine inlet temperature (TIT)	Turbine exit pressure (TEP)	Mass flow rate of molten salt (ṁ)	Direct normal irradiation (DNI)
A	Exergy Efficiency	4	4	4	2	5
B	Energy Efficiency	3	5	3	2	5
C	Ecological Efficiency	2	3	3	1	4
D	Economic Efficiency	3	4	3	1	5

The vital weightage allocated to separately criterions in linguistic counterparts were autonomously estimated by a decision-management participant of the powerplants.

Table 7 establishes the input based combined weightages achieved from different problems. In order to create the optimum formulation, a homogenous weighting feature is used.

**Table 7:** Combined weights project procedure by participants of the study.

Identification	Weightage	Criterion
C1	Turbine inlet pressure (TIP)	22.6%
C2	Turbine inlet temperature (TIT)	29.5%
C3	Turbine exit pressure (TEP)	10.1%
C4	Mass flow rate of molten salt (ṁ)	5.6%
C5	Direct normal irradiation (DNI)	32.2%

### Outcome parameters

The study of solar cogeneration in a Rankine cycle involves analysing several outcome parameters, including Exergy Efficiency, Energy Efficiency, Ecological Efficiency, and Economic Efficiency. Following are the importance of each outcome parameter:

#### Exergy efficiency

Exergy Efficiency measures the ratio of useful work output to the maximum potential work output of a system. It provides a comprehensive assessment of the quality of energy conversion within the system, considering both the quantity and the quality of energy. Maximizing Exergy Efficiency in a solar cogeneration Rankine cycle ensures optimal utilization of available energy and minimizes energy losses, leading to improved overall system performance and sustainability.

#### Energy efficiency

Energy Efficiency focuses on the ratio of useful energy output to the total energy input in the system. It quantifies how effectively energy is converted into useful work or output. High Energy Efficiency indicates a more sustainable and economical operation, as it minimizes energy waste and reduces the resource consumption. Enhancing Energy Efficiency in a solar cogeneration Rankine cycle results in increased power generation capacity, reduced environmental impact, and improved energy management.

#### Ecological efficiency

Ecological Efficiency assesses the environmental impact of the system by considering factors such as greenhouse gas emissions, pollutant discharge, and resource depletion. It measures the system's ability to utilize energy resources in an environmentally friendly manner. A high Ecological Efficiency in a solar cogeneration Rankine cycle demonstrates the system's capability to minimize negative environmental impacts, contribute to sustainable development, and support a cleaner and greener energy transition.

#### Economic efficiency

Economic Efficiency evaluates the financial viability and profitability of the system. It considers factors such as capital investment, operating costs, revenue generation, and the overall economic benefits. Achieving high Economic Efficiency in a solar cogeneration Rankine cycle ensures a cost-effective utilization of resources and demonstrates the system's economic competitiveness. It supports the feasibility and attractiveness of solar cogeneration projects by demonstrating their financial viability and long-term sustainability.

Considering and optimizing Exergy Efficiency, Energy Efficiency, Ecological Efficiency, and Economic Efficiency in the study of solar cogeneration in a Rankine cycle are vital for achieving sustainable energy utilization, minimizing environmental impact, and ensuring economic viability. By focusing on these outcome parameters, researchers and engineers can design and operate solar cogeneration systems that are efficient, environmentally friendly, and economically feasible.

## Results and discussions for the proposed system

The following important parameters have been analysed such as: the effect of operating parameters on different efficiencies of the system such as DNI, a mass flow rate of molten salt and steam, turbine inlet pressure, turbine efficiencies of the inlet, and exergy degradation. Thereafter the thermodynamic properties of refrigerants such as R-113, R-11, and R-1233zd have been examined in the ORC cycle, and the results have been validated.

### Effects of direct normal irradiation (DNI) on system

**Figure 4:**
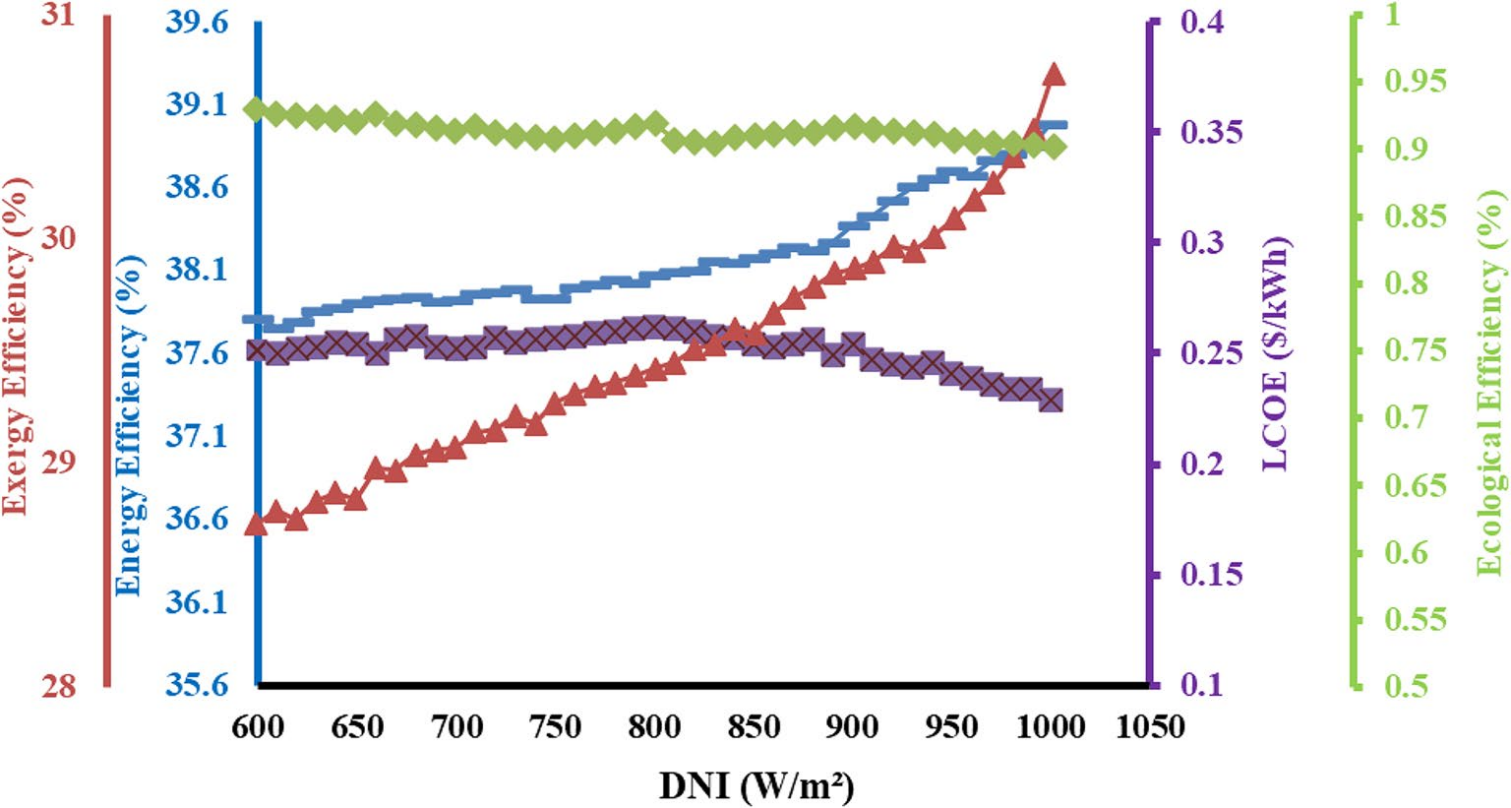
Impact of DNI on efficiencies of the proposed system.

Direct normal irradiation plays a vital role in 4-E analysis since the proposed system primary energy generation is with the aid of solar equipment. The overall performance of the proposed setup is highly dependent on DNI, which varies with the different time of each month and day. Moreover, DNI impacts the mass flow rate of the proposed system. From the analysis it is apparent that mass flow rate of molten salt increases gradually with constant rise in DNI value. This further translates into an increase in mass flow rate of the steam circulated in the cogeneration system, thereby increasing the energy generation rate. As the DNI value is varied and incremented from 600 W/m^2^ to 1000 W/m^2^, a rapid gain in exergy is registered which is probably due to more energy available at inlet which is dissipated in the various components of the cycle evidently displayed in Fig. 4. This is also validated from previous studies where an increment in DNI value increases, both the energy and exergy efficiencies of the hybrid setup, simultaneously accompanying with nearly linear rise of the power production capacity^41,42^. As the condensate waste heat is completely recovered in cogeneration plant, the energy efficiency can reach up to 78% approximately. Furthermore, the cost of the system also decreases as the electricity generation capacity increases which provides added profits to the system. At lower DNI value, the exergy efficiency is lower due to inferior quality of energy availability present at lower temperature. The exergy efficiency is dominated by central receiver and steam turbine where best possible energy is transferred through molten salt. While the hybrid setup generates more power with the increasing DNI value. For example when the DNI value is only 600 W/m^2^, the power production efficiency is as small as 37.8%. while at 1000W/m^2^ the efficiency goes as high as 39.1% for a constant mass flow rate of molten salt. Therefore, the overall exergy efficiency can reach the highest value of 39.8%. The exergy efficiency of 39.8% in integrated novel system is smaller than 45.35% of STP tower plant. When energy of the system increases, the ecological efficiency decreases, with a simultaneous decrease in LCOE of the system. EE is inversely proportional to energy of the system and LCOE is on the contrary^33^. When energy production is larger than LCOE of the setup is lower. At 1000 W/m^2^ the cost of electricity production is lowest at 0.23 $/unit, with minimum environmental impact on the atmosphere. It is also noted that the entire cycle efficiency of the exergy analysis is less than the energy observed in the combined heating and power cogeneration energy system; it is because of the major quantity of exergy associated with the thermal energy which has been received less than its energy content. It has been observed that energy output significantly enhanced with a subsequent variation in DNI, it founds between 3700 and 6400 kW when the DNI is varied between 600 W/m^2^ and 1000 W/m^2^. The causes of that rising trend is the significantly increasing the process heat output. It has been observed that exergy output of cycle increases but the magnitude of total exergy output increment is observed quite less than its corresponding energy output. This deviation is observed due to the reason the exergy accompanied by process heat output is significantly less than their corresponding energy output since the magnitude of electrical power gives a 100% of exergy is increasing at a lower rate than the increase of their exergy associated with process heat. Therefore, the overall exergy output is increasing at all values of DNI less than the overall exergy cycle of output. The similar results are presented by previous study^32^ it means the presented system of this communication is apparently validated.

### Effect of turbine inlet temperature (TIT) on system

**Figure 5:**
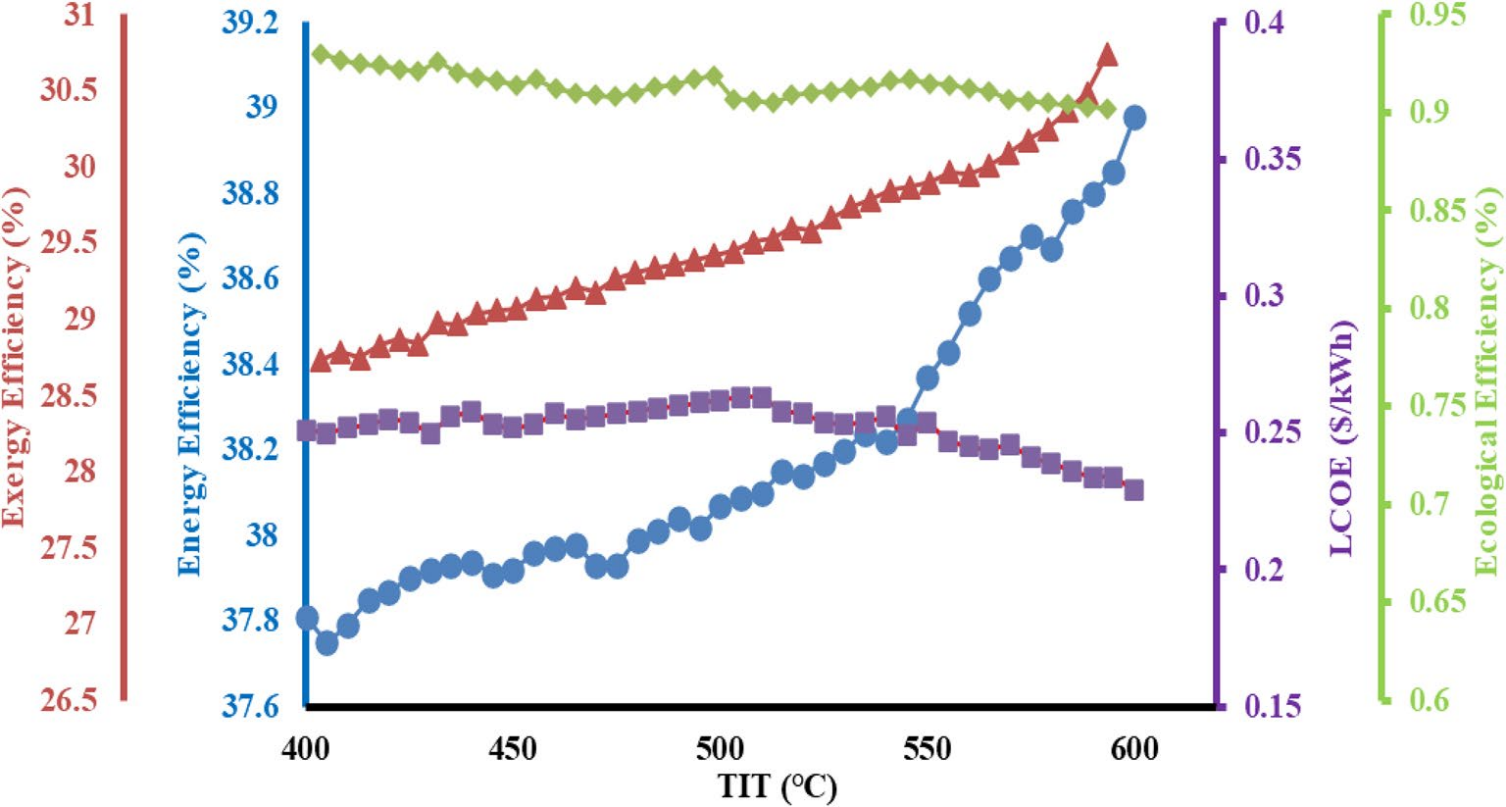
Impact of TIT on efficiencies of the proposed system.

Figure 5 expressed the variations of the thermodynamic and economic efficiencies of the entire system performance with the variation in the turbine inlet temperature (TIT) for combined heating and power (CHP). It is observed that the thermodynamic efficiencies of the entire system increase appreciably with an increase in TIT. It is also observed that the increasing the TIT will be increasing the power output from the steam turbine cycle. In particular, exergy efficiency rises proportionally by increasing the inlet temperature of turbine, which is primarily attributed due to the inlet available energy to the cycle which remains constant^43^. This further translates to a gradual increase in the power output, eventually increasing the exergy efficiency also rises. The energy efficiency in accordance with exergy efficiency also increases due to an increment in the thermal efficiency of the system which is responsible for a rise in the inlet pressure to the turbine. The results are justifying the benefits of the integration of user heat (UH), ORC and RC, which shows a significant increment in both the efficiencies of the combined heating and power of cogeneration energy system^44,45^. It is observed that the TIT is an important parameter in the design of the solar-operated organic Rankine cycle. The LCOE of the system first increases linearly and then rapidly falls since the increment in the evaporation temperature of the refrigerant, decreases the mass flow rate. This eventually translates into, an increase in the pressure which further increases the final temperature of the evaporator and condenser. Simultaneous action of reduction in mass flow rate and increase in temperature of evaporator and condenser decreases the LCOE of the system. Increase in thermal efficiency of the system results in decreased ecological efficiency thereby emitting lower pollutants.

### Effect of steam turbine inlet pressure (TIP) on system

**Figure 6:**
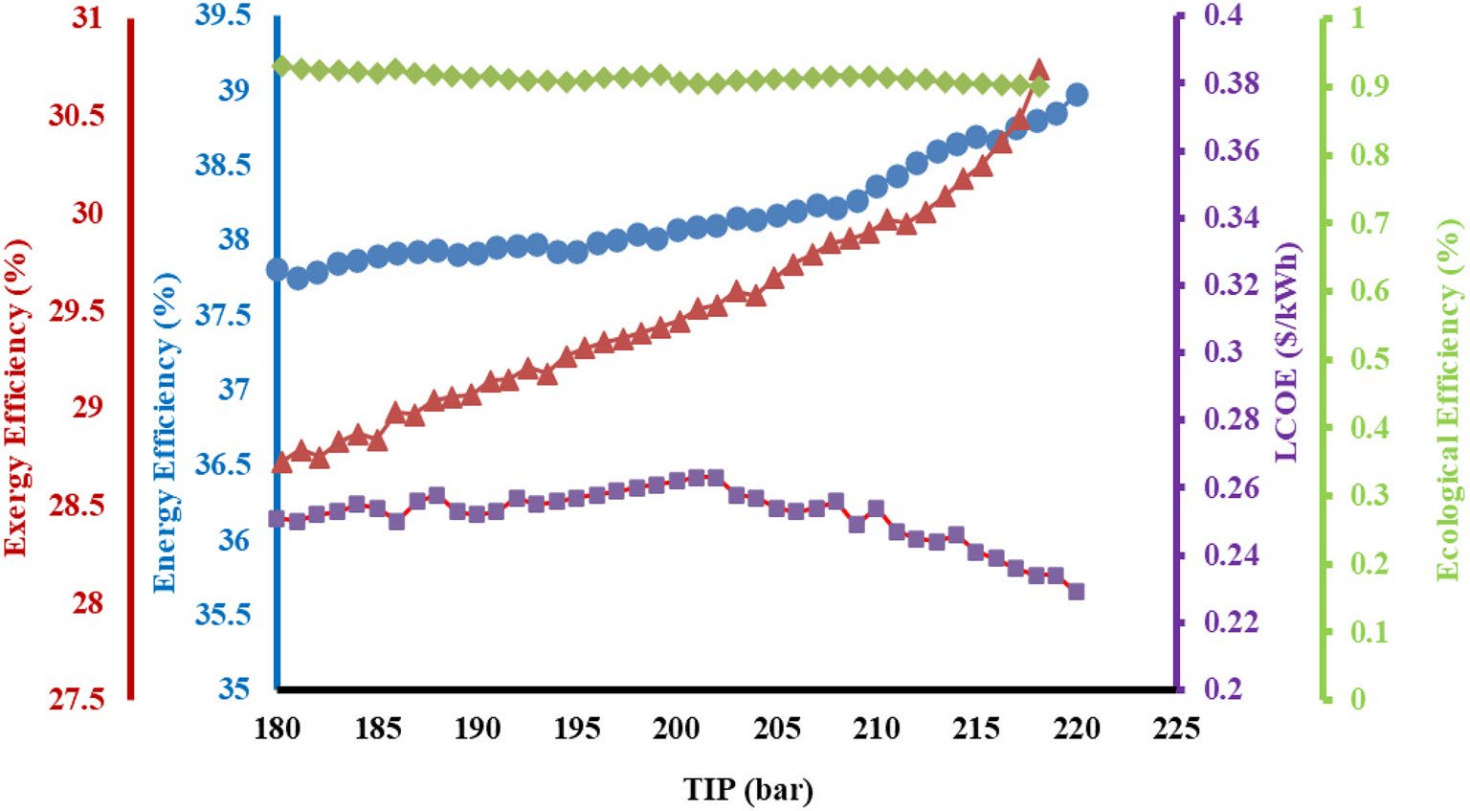
Impact of TIP on efficiencies of the proposed system.

The effect of steam turbine entrance pressure (TIP) on the entire energy and exergy efficiencies is shown in Fig. 6. It is observed that, both the thermodynamic (energy and exergy) efficiencies of the system being rise with increasing TIP value. The energy available at inlet increases which eventually increases the power produced by the turbine, yielding superior energy efficiency. While, the exergy efficiency increases in almost linear fashion since the power output in the ORC turbine cycle increases, eventually increasing the power output of the steam turbine cycle^46^. Therefore, the entire primary efficiency of the system will be increasing. Moreover, the proportion of increment in the heating cycle yield of ORC is significantly higher, which ultimately increases the overall power output generated through the steam turbine cycle^47,50^. It found that the incremental behaviour of the exergy efficiency is slightly less than the energy efficiency at the same pressure. It has occurred due to the exergy in the related heating system is lower than the energy generated through process heat^48,51^. Furthermore, higher power output for same mass flow rate decreases the LCOE of the system with minimum value of 0.24 $/unit of electricity achieved at 220 bar. Higher energy efficiency makes the system more energy efficient, thereby emitting lower amount of pollutants. Additionally, the lower pump requirement due to decrease in mass flow rate lowers the environmental impact to only 0.85 at 220 bar.

### Effect of the Turbine exit pressure (TEP) on system

**Figure 7:**
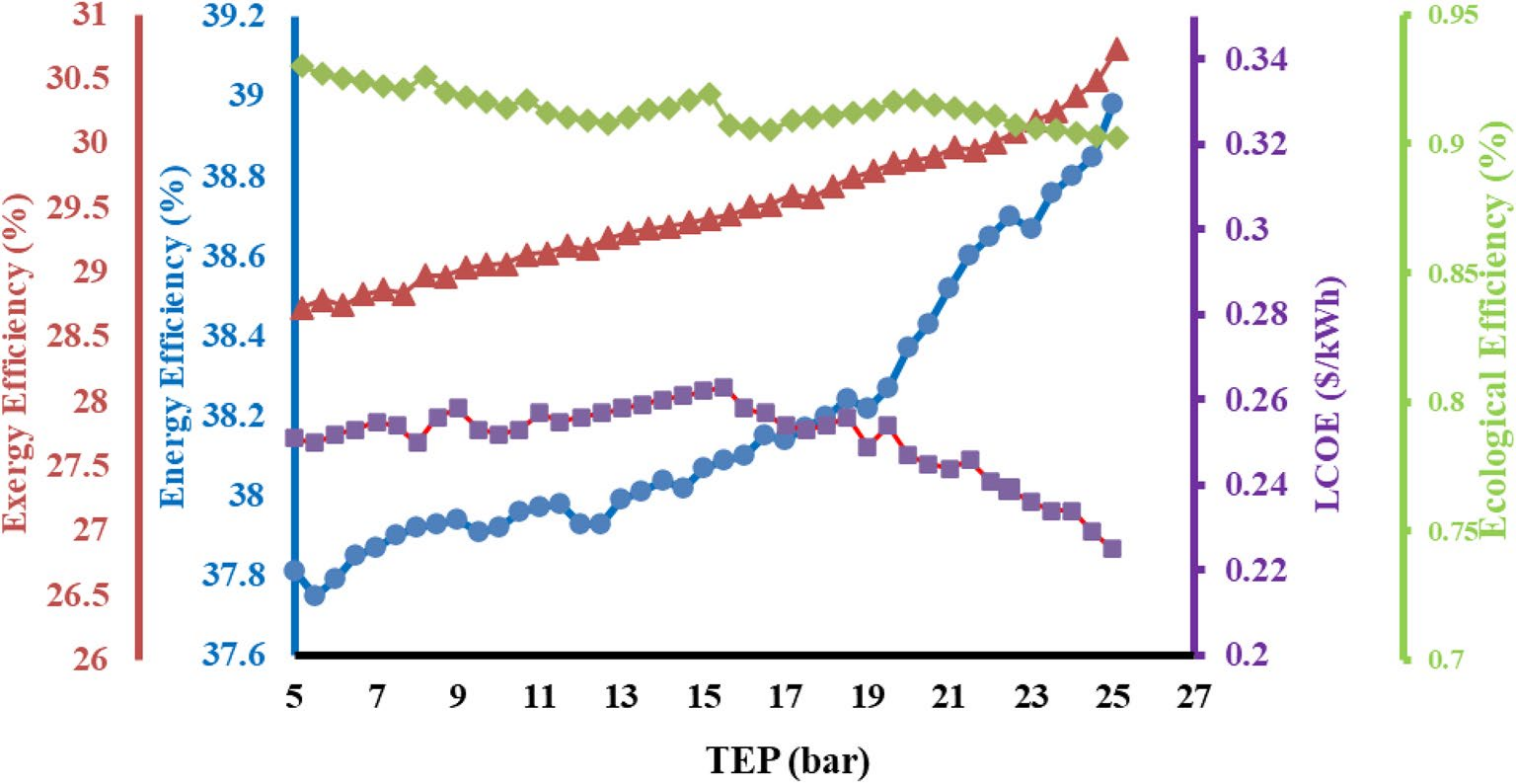
Impact of TEP on efficiencies of the proposed system.

The effect of the pressure of the steam leaving the turbine through the heater on energy and exergy efficiencies is shown in Fig. 7. While increasing the pressure at the outlet of the turbine from 5 to 27, translates into more steam pressure available at inlet, eventually resulting in a marginal rise in the energy and exergy efficiencies of the system^48,52^. An almost linear gain in the energy efficiency of the system is displayed, since more pressure available at inlet produces more power at inlet by the system. Furthermore, a better and superior quality of steam is available to be transformed into mechanical work. Utmost care should be taken to attain better steam with higher temperatures since inferior quality of steam results in lower temperatures and lower energy transformation^49^. It is also interpreted that the exergy destruction will be increasing in the heater when thermal energy is transferred between working fluids at a point where the temperature difference is observed maximum. Conversely, steam obtained at low pressures results in enhanced work output within the turbine due to the enthalpy drop increases. Higher temperatures attained for steam results in lower EE value which is contrary to LCOE. Higher turbine pressures give surplus energy to cogeneration system resulting in lower cost of electricity. These trends are validated by previous works also^41,47,53^.

### Effect of variation of refrigerants like R-113, R-11, and R-1233zd on thermal performance of ORC

**Figure 8:**
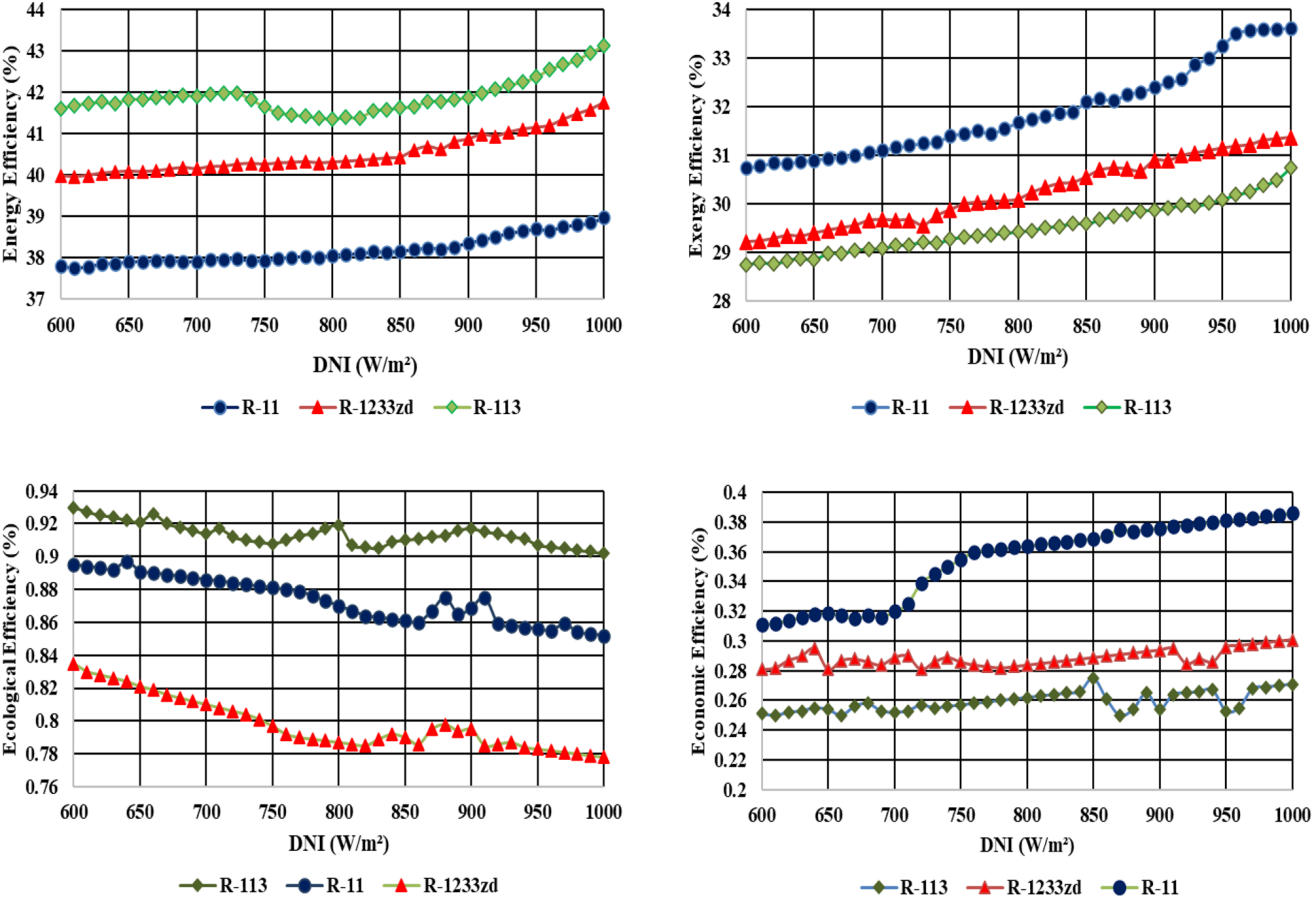
4-E comparison of the three test working fluids based on DNI.

The effect of variation of refrigerants like R-113, R-11, and R-1233zd on thermodynamic performance of ORC like thermodynamic (energy and exergy) efficiencies with the increase in the TIT of ORC, which is shown in Fig. 8. The trends of higher efficiencies observed for all the working fluids for all TIP variations from 110 to 190 °C. The subsequently increasing the efficiencies, primarily due to surplus electric power generation which is attributed the expansion of working fluid in the ORC turbine. Since DNI is estimated the most significant parameter, hence the refrigerants are compared by varying DNI value. It should be the bare minimum. Notable add-on efficiency is observed when employing of R-113. It as a heat transfer fluid (refrigerant) used in the ORC. Concurrently, the R-11 fluid appears superior efficiency than R**-**1233zd**.** Comparing the results, it should be noted that R-113 has a higher boiling point than R-11 and further its boiling is higher than R-1233zd and the mark-able efficiencies observed of these three fluids in the queue like ${{\varvec{\eta}}}_{{{\varvec{R}}}_{113}}>{{\varvec{\eta}}}_{{{\varvec{R}}}_{11}}>{{\varvec{\eta}}}_{{{\varvec{R}}}_{1233{\varvec{z}}{\varvec{d}}}}$,these results are validated the results of previous studies^23,40^.

### Weightage analysis

Analytical hierarchy process (AHP) is an important tool in generating priority and weightage for multiple constraints or criterions of the system. It comprises of an ability to transform a complex problem into simpler ones by identifying primary constraints and provide them with special weights to balance the system similar to real world problems. In this problem multiple variables were selected for analysis through previous literature on similar topics. But it was difficult to comprehend that which parameter has more impact than other. Henceforth, AHP is applied to estimate the weightages. The AHP is accountable for conveying weights to multiple criterions. Moreover, the outcomes of the system are ranked and rated from based on previous literatures^43,44,54^. The values are expressed in linguistic terms in accordance to their weightages to obtain a balanced problem statement as portrayed in Table 8.

**Table 8:** Weights assigned to the attributes of various models.

S.No	Outcomes	Turbine inlet pressure (TIP)	Turbine inlet temperature (TIT)	Turbine exit pressure (TEP)	Mass flow rate of molten salt (ṁ)	Direct normal irradiation (DNI)
Weightages		**0.226**	**0.295**	**0.101**	**0.056**	**0.322**
Importance Criteria Rank		1	2	4	3	5
A	Exergy Efficiency	5	4	2	5	3
B	Energy Efficiency	1	2	2	4	2
C	Ecological Efficiency	3	3	5	4	1
D	Economic Efficiency	4	5	5	3	5

Significant values are in bold.

**Figure 9:**
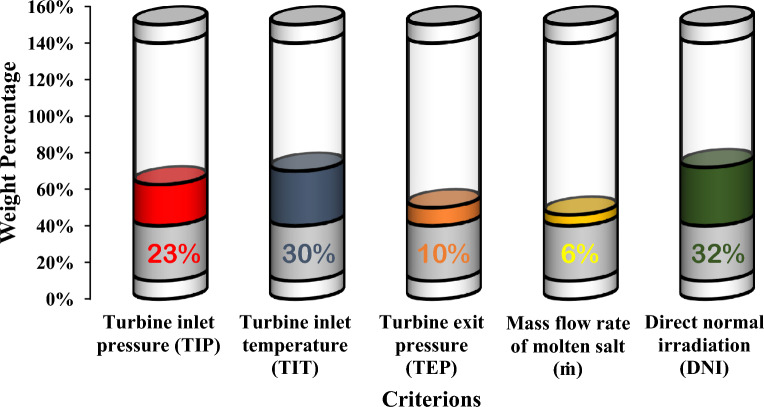
Influence of multiple criterions on 4-E analysis.

It is quite evident from Table 8 that attaining an optimum balance among operating parameters is essential as the weights achieved vary substantially from each other. Application of signifies global weights to criterions, prepared in a tabular form by specific weights to operating parameter based on the attained value as shown in Fig. 9.

The universal importance weights of the criteria's along with their rankings are established in Table 8. From the investigation it is found that supreme weight or importance is achieved in case of DNI value criterion (32%) as compared to other parameters, while mass flow rate achieves lowest importance (6%).

### TOPSIS analysis

Often any system is judged on the basis of its final outcomes. But some outcomes are more important to the user than others, which is why a priority score needs to be developed on the basis of multiple operating characterises^44^. TOPSIS analysis is capable of identifying those outcomes which are essential for user in comparison to other outcomes. This preference changes from country to country and season to season. The performance outcomes were prioritized with the aid of a single performance score through TOPSIS-AHP methods. AHP technique, as already explained above, is applied to estimate the weightages of operating parameters. The weights achieved above, are applied in TOPSIS model to calculate a single performance score for the four outcomes.

In the meantime, the elements of the performance attributes remained fairly divergent, henceforth they reallocated to be standardized to convert them into a equivalent array in linguistic relationships.

The performance score of individual outcomes was considered after investigating the optimal best and optimal worst. In addition, Euclidean distance in individual stages is revealed in Table 9.

**Table 9:** Positive and negative ideal solution matrix along with performance score.

S.No	Outcomes	Turbine inlet pressure (TIP)	Turbine inlet temperature (TIT)	Turbine exit pressure (TEP)	Mass flow rate of molten salt (ṁ)	Direct normal irradiation (DNI)	P_i_ Score
Weightages		**0.226**	**0.295**	**0.101**	**0.056**	**0.322**	
Importance criteria rank		3	2	4	5	1	
A	Exergy efficiency	0.10	0.09	0.02	0.07	0.03	0.49
B	Energy efficiency	0.02	0.05	0.02	0.06	0.02	0.53
C	Ecological efficiency	0.06	0.07	0.04	0.06	0.01	0.23
D	Economic efficiency	0.08	0.12	0.04	0.04	0.05	0.26

Finally, Table 10 shows the outcomes ranking and importance in this hybrid system.

**Table 10:** Ranking of outcomes among 4-E analysis.

Outcomes	P_i_ (Score)	Rank
Exergy efficiency	0.49	2
Energy efficiency	0.53	1
Ecological efficiency	0.23	4
Economic efficiency	0.26	3

### Exergy destruction for all major components of the proposed system

**Figure 10:**
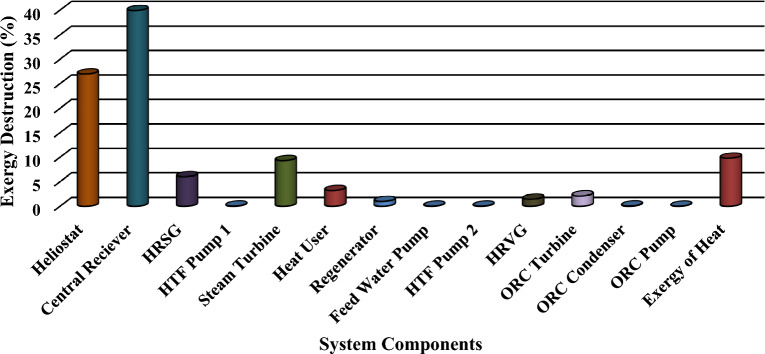
Exergy destruction in each component in the cogeneration energy system.

Figure 10 shows the distribution of exergy destruction for all major components in the system. It has been evaluated that the central receiver and heliostat have the largest exergy destruction due to the larger temperature difference in the system about 39.92% and 27%, respectively. A substantial quantity of exergy destruction is noticed in the steam turbine around 9.32%. Among the other components incorporated in the model, HU estimated combined exergy destruction of 3.2% examined on an individual basis. This exergy destruction analysis provides useful data and also aids the designer to generate the ranking among components development for the proposed solar-operated cogeneration energy system and the results are approved with the similar results of previous investigation^44,50,55^.

### Validation of proposed model with previous literature

To validate the findings of our study on the percentage exergy loss in solar cogeneration Rankine cycle, we conducted a rigorous analysis and followed established methodologies. Table 11 provides a comparative analysis of various components exergy losses used in prior studies and the current study. The inclusion of different studies multiple approaches with various models helps establish a broader context for the current study. This comparison aids in understanding the uniqueness and contribution of the current study, which utilizes the combined TOPSIS-AHP method. It provides researchers and readers with valuable insights into the existing body of knowledge and highlights the specific focus and novelty of the current study. The validation process ensures the credibility and robustness of our findings, providing confidence in the conclusions drawn from our research.

**Table 11:** Comparison of exergy loss in different models with prior studies.

References	Model	Component	Exergy loss
Oyekale et al.^47^	Solar-Biomass ORC-cogeneration	Central field	51.56%
Kerme et al.^48^	Solar-Desalination poly-generation	Thermal collector	41.32%
Ding et al.^49^	Solar heat- ORC	Storage system	44.98%
Current study	Solar-ORC-cogeneration	Central field	39.92%

## Academic contribution of the study

Our present study makes significant academic contributions in the field of solar cogeneration and exergy analysis. Firstly, we address several research gaps identified in the available literature. These include the integration of the ORC cycle with the heliostat field's solar collector for combined heating and power generation, which has been rarely studied before. By examining an innovative cascade solar-thermal arrangement, we efficiently recover waste heat from the steam turbine, resulting in enhanced system performance. Additionally, we conduct an in-depth thermodynamic investigation, considering factors such as exergy losses, energy efficiencies, and thermodynamic performance parameters. This detailed analysis provides valuable insights into the operational characteristics and performance optimization of the solar cogeneration Rankine cycle. Furthermore, we provide an economic analysis by evaluating net electricity generated per unit, assessing the economic feasibility and potential benefits of the proposed system. Lastly, we explore the system's environmental impact by assessing its effluents and ecological effectiveness, contributing to the understanding of its sustainability and potential for reducing greenhouse gas emissions.

In conclusion, our study fills significant research gaps in the field of solar cogeneration by integrating the ORC cycle with the heliostat field's solar collector and conducting an enhanced thermodynamic investigation. Furthermore, we provide economic and environmental analyses, offering insights into the feasibility, benefits, and sustainability of the system. These contributions expand the knowledge base in the field, guide future research endeavours, and inform decision-making processes in solar cogeneration and related areas.

## Conclusions

The exergy analysis has been received global positive response over the traditional energy procedures and found acceptance by both academia and industry simultaneously. The present analysis has been established the functional conditions for a solar-operated steam turbine to HU and also recover the waste heat while ORC is integrated at the bottom of a steam generator. Keeping the thermodynamic point of view consideration to the integration of the Rankine cycle, HU and organic Rankine cycle while considering the heat rejected of the steam cycle as waste heat provides better efficiency because of the waste heat is utilized for additional heating and power generation. In addition, uncertainty is also introduced to obtain accurate results with a precision that should be removing all human and machine errors in the current study. Following concluding remarks are coined from the research below:

- A slight increase in the energy and exergy efficiencies has been observed after a considerable increase in the DNI at different operating conditions.
- The energy efficiency at mean operating conditions of DNI have been observed in the range of 40.61% to 41.74% and the exergy efficiency evaluated in the range of 30.74% to 31.84% when the ORC being employed to produce power.
- While TIT increases, slightly increases the Energy and exergy efficiencies for the combined heating and power cogeneration energy system.
- The energy efficiency has been increases significantly while the TIP increases, whereas the exergy efficiency of a combined heating and power cycle increases slightly with the same operating condition.
- The most significant parameter which impact the performance of the system is DNI (32%), while least impact is of mass flow rate (6%).
- A significant increase in the mass flow rate of molten salt and the mass flow rate of steam has been increased with increasing the DNI values.
- By the consideration of mean operating conditions, it is observed that R-113 best results, afterward, R-11 being the rank second and R-1233zd obtained the rank third used in the ORC cycle respectively.
- Out of 100% exergy input of the cycle, the highest exergy destruction is found to be estimated 39.92% in the central receiver, 27% in heliostat, and 9.32% in a steam generator, 3.2% in the HU, and 3.633% in ORC cycle, etc.

The research and model have a few limitations which can be analysed for future scope. A sensitivity analysis is required for the identified parameters, yet the extent of their impact and sensitivity thresholds remain unexplored. Moreover, the research does not adequately address equipment uncertainty and environmental factors, necessitating a more comprehensive examination of these aspects to enhance the research's accuracy. Also, the study acknowledges the limitation of Multi-Criteria Decision Making (MCDM) to only seven input criteria, beyond which it produces errors, limiting its applicability to complex systems.

Future researches can focus on enhancing system performance through advanced control strategies, improved component design, and integration of emerging technologies. The integration of energy storage systems can enable better utilization of intermittent solar energy, ensuring a continuous and reliable power supply. Additionally, exploring novel refrigerants, advanced heat transfer fluids, and optimized thermal storage solutions can further enhance energy conversion efficiency and overall system sustainability. In the near future, the parameters identified in this study can undergo a thorough critical analysis through a comprehensive sensitivity analysis of the entire setup. This analysis will contribute to the validation of the performance outcomes. The timing of both charging and discharging plays a crucial role in the system's storage capabilities. Consequently, there is a need for more focused future research to determine the optimal timing parameters for the current system.

Furthermore, the integration of smart grid technologies, grid-friendly operation strategies, and the exploration of hybrid solar cogeneration systems can maximize system flexibility and contribute to the efficient integration of solar cogeneration into existing energy infrastructure. Continued advancements in these areas will pave the way for widespread adoption of solar cogeneration in the future, enabling clean, efficient, and sustainable energy generation and meeting the growing energy demands of the world.

### Supplementary Information

Supplementary Information.

## Data Availability

The datasets used and/or analysed during the current study available from the corresponding author on reasonable request.

## Abbreviations

$${A}_{{\rm{app}}}$$: Aperture area (m^2^)
${\dot{A }}_{{\rm{field}}}$: Area of heliostat field (m^2^)
DNI: Direct normal irradiation
$$ \dot{E} $$: Exergy (kW)
Exergy destruction (kW)
HRSG: Heat recovery steam generator
HRVG: Heat recovery vapour generator
h: Enthalpy (kJ/Kg)
$\dot{m}$: Mass flow rate (Kg/sec)
${\dot{m}}_{{\rm{vapour}}}$: Mass flow rate of vapour (Kg/sec)
${\dot{m}}_{wt}$: Mass flow rate of water (Kg/sec)
${\dot{m}}_{g}$: Mass flow rate of refrigerant (Kg/sec)
${\dot{m}}_{st}$: Mass flow rate of steam (Kg/sec)
${\dot{m}}_{{\rm{moltensalt}}}$: Mass flow rate of molten salt (Kg/sec)
ORC: Organic Rankine cycle
q: Amount of solar radiation per unit area (W/m^2^)
${{\dot{Q}}}_{{\text{A}}}$: Energy of the absorber (kW)
${\dot{Q}}_{CR}$: Heat energy of the central receiver (kW)
${{\dot{Q}}}_{{\text{E}}}$: Energy of the evaporator (kW)
${\dot{Q}}_{{\rm{lost}},\mathrm{ CR}}$: Loss of heat energy from the central receiver (kW)
${\dot{ Q}}_{{\rm{lost}},\mathrm{ heliostat}}$: Loss of heat energy from heliostat (kW)
${\dot{Q}}_{{\rm{salt}}}$: Heat energy of molten salt (kW)
${\dot{Q}}_{{\rm{solar}}}$: Heat energy of solar (kW)
RC: Rankine cycle
RT: Refrigerant turbine
s: Specific entropy (kJ/K)
$${T}_{0}$$: Ambient temperature (^o^C)
${\dot{W}}_{P}$: Pump work (kW)
${\dot{W}}_{RT}$: Refrigerant turbine work (kW)
${\dot{W}}_{T}$: Turbine work (kW)
$${\eta }_{{\rm{energy}}}$$: Energy efficiency
$${\eta }_{{\rm{energy}}}$$: Exergy efficiency
${\eta}_{{\rm{energy}},\mathrm{ heliostat}}$: Energy efficiency of heliostat
a–d: State points of a cogeneration system
1–17: State points a cogeneration energy system
