# Supplementary material for: 4-E analysis and multiple objective optimizations of a novel solar-powered cogeneration energy system for the simultaneous production of electrical power and heating
Source: Sci Rep. 2023 Dec 14;13:22246. doi: 10.1038/s41598-023-49344-2 (PMC10721817; doi:10.1038/s41598-023-49344-2)
Supplement: Supplementary file 1 — Supplementary Information. [file 41598_2023_49344_MOESM1_ESM.docx]

**Supplementary Section**

Table A1: Energy balance equation of each component for the solar-powered cogeneration energy system

| **Component** | **Energy Balance Equations** |
| --- | --- |
| **Cogeneration Energy System** | |
| **Central Receiver** | $\dot{Q}_{CR}=\dot{Q}_{salt}+\dot{Q}_{lost, CR}=\dot{m}_{moltensalt}\left( h_{1}-h_{3} \right)+\dot{Q}_{lost, CR}$ |
| **HRSG** | $\dot{m}_{molten salt}\left( h_{1}-h_{2} \right)=\dot{m}_{st}\left( h_{4}-h_{10} \right)+\dot{m}_{steam}\left( h_{6}-h_{5} \right)$ |
| **Steam Turbine** | $\dot{W}_{ST}=\dot{m}_{st}\left[ \left( h_{4}-h_{5} \right)+\left( h_{6}-h_{7} \right) \right]+ (1-y)\dot{m}_{st}(h_{7}-h_{11})$ |
| **Heat User** | $\dot{m}_{st}y\left( h_{7}-h_{8} \right)=\dot{m}_{hw}\left( h_{b}-h_{a} \right)$ |
| **HTF Pump 1** | $\dot{W}_{HTFP1}=\dot{m}_{molten salt}\left( h_{3}-h_{2} \right)$ |
| HTF Pump 2 | $\dot{W}_{HTFP2}=\left( 1-y \right)\dot{m}_{st}\left( h_{13}-h_{12} \right)$ |
| **Regenerator** | $\dot{m}_{st}{yh}_{8}+\left( 1-y \right)\dot{m}_{st}h_{13}=\dot{m}_{st}h_{9}$ |
| **Feed Water Pump 3** | $\dot{W}_{FWP3}=\left( 1-y \right)\dot{m}_{wt}\left( h_{10}-h_{9} \right)$ |
| **HRVG** | $\dot{m}_{st}\left( h_{11}-h_{12} \right)=\dot{m}_{g}\left( h_{17}-h_{14} \right)$ |
| **ORC Turbine** | $\dot{W}_{ORCT}=\dot{m}_{g}\left( h_{14}+h_{15} \right)$ |
| **ORC Condenser** | $\dot{m}_{g}\left( h_{15}-h_{16} \right)=\dot{m}_{f}\left( h_{d}-h_{c} \right)$ |
| **ORC Pump 4** | $\dot{W}_{ORCP4}=\dot{m}_{f}\left( h_{17}-h_{16} \right)$ |

Table A2: Exergy balance equation of each component for the solar-powered cogeneration energy system

| **Component** | **Exergy Balance Equations** | |
| --- | --- | --- |
| **Cogeneration Exergy System** | | |
| **HRSG** | | ${\dot{E}_{D, HRSG}=T}_{0}\left[ \dot{m}_{molten salt}\left( s_{2}-s_{1} \right)-\dot{m}_{st}\left( s_{4}-s_{10} \right)-\dot{m}_{st}\left( s_{6}-s_{5} \right) \right]$ |
| **Steam Turbine** | | $\dot{E}_{D,ST}=\dot{m}_{st}T_{0}\left[ \left( s_{5}-s_{4} \right)+s_{7}y+\left( 1-y \right)\left( s_{11}-s_{6} \right) \right]$ |
| **Heat User** | | $\dot{E}_{D,HU}=T_{0}\left[ \dot{m}_{st}y\left( s_{8}-s_{7} \right)+\dot{m}_{hw}\left( s_{b}-s_{a} \right) \right]$ |
| **HTF Pump 1** | | $\dot{E}_{D,HTFP1}=T_{0}\dot{m}_{molten salt}\left( s_{3}-s_{2} \right)$ |
| HTF Pump2 | | $\dot{E}_{D,HTFP2}=T_{0}\dot{m}_{wt}\left[ \left( 1-y \right)\left( s_{13}-s_{12} \right) \right]$ |
| **Regenerator** | | $\dot{E}_{D, Reg}=T_{0}\dot{m}_{st}\left[ \left( s_{9}-{ys}_{8} \right)-\left( 1-y \right)s_{13} \right]$ |
| **Feed Water Pump 3** | | $\dot{E}_{D,FWP3}=T_{0}\dot{m}_{wt}\left[ \left( 1-y \right)\left( s_{10}-s_{9} \right) \right]$ |
| **HRVG** | | $\dot{E}_{D, HRVG}=T_{0}\left[ \dot{m}_{st}\left( s_{11}-s_{12} \right)+\left( s_{17}-s_{14} \right) \right]$ |
| **ORC Turbine** | | $\dot{E}_{D,ORCT}=T_{0}\left[ \dot{m}_{g}\left( s_{14}-s_{15} \right) \right]$ |
| **ORC Condenser** | | $\dot{E}_{D,ORCC}=T_{0}\left[ \dot{m}_{g}\left( s_{15}-s_{16} \right)+\dot{m}_{f}\left( s_{d}-s_{c} \right) \right]$ |
| **ORC Pump 4** | | $\dot{E}_{D,OPECP4}=T_{0}\left[ \dot{m}_{f}\left( s_{17}-s_{16} \right) \right]$ |
